# Retrospective Evaluation of a Single Surgeon’s Learning Curve of Robot-Assisted Radical Cystectomy with Intracorporeal Urinary Diversion via Ileal Conduit

**DOI:** 10.3390/cancers15153799

**Published:** 2023-07-26

**Authors:** Christof Achermann, Andreas Sauer, Marco Cattaneo, Jochen Walz, Stephen F. Wyler, Maciej Kwiatkowski, Lukas W. Prause

**Affiliations:** 1 Department of Urology, Cantonal Hospital Aarau, 5001 Aarau, Switzerland; 2 Department of Urology, University Hospital of Basel, University of Basel, 4001 Basel, Switzerland; 3 Department of Clinical Research, University of Basel, 4001 Basel, Switzerland; 4 Department of Urology, Institut Paoli-Calmettes Cancer Center, 13009 Marseille, France; 5 Medical Faculty, University of Basel, 4056 Basel, Switzerland; 6 Department of Urology, Academic Hospital Braunschweig, 38126 Braunschweig, Germany

**Keywords:** learning curve, robotics, radical cystectomy, intracorporeal urinary diversion, bladder cancer

## Abstract

**Simple Summary:**

Robot-assisted surgery for removal of the bladder is becoming more common. In recent years, the technique has emerged to allow the entire procedure to be performed within the abdominal cavity, without requiring a conversion to open surgery. However, especially the reconstructive part of the procedure can be difficult for surgeons to learn. Our center started using this technique in 2017, and we evaluated the first 53 cases, all performed by one surgeon, to see how the surgeon’s experience affected the result. We found that, as the surgeon gained more experience with the technique, the procedure was performed faster and had fewer complications. Our analysis shows that complex cystectomies should be performed by surgeons who had experience with more than 40 cases.

**Abstract:**

Robot-assisted radical cystectomy with intracorporeal urinary diversion (iRARC) is increasingly being performed instead of open surgery. A criticism of this technique is the long learning curve, but limited data are available on this topic. At our center, the transition from open radical cystectomy (ORC) to iRARC began in May 2017. A retrospective analysis was conducted on the initial 53 cases of robot-assisted cystectomy with intracorporeal urinary diversion via ileal conduit, which were performed by one single surgeon. The patients were divided into four consecutive groups according to the surgeon’s increasing experience, and perioperative parameters were analyzed as a surrogate for the learning curve. Over the course of the learning curve, a decline in median operation time from 415 to 361 min (*p* = 0.02), blood loss from 400 to 200 mL (*p* = 0.01), and minor complications from 71% to 15% (*p* = 0.02) was observed. No significant difference in overall and major complications, length of hospital stay, and total lymph node yield was shown. During the initial period of the learning curve, only the less complex cases were operated on using robotic surgery, while the more challenging ones were handled through open surgery. After experience with 28 cases, no more cystectomies were performed through open surgery. This led to an increase in operation time and length of hospital stay, as well as a higher incidence of both minor and overall complications among cases 28–40. After 40 cases, a significant decrease in these parameters was observed again. Our analysis demonstrated that operation time, blood loss, and minor complications decrease with increasing surgical experience in iRARC, while suggesting that technically challenging cases should be operated on after experience with 40 robotic cystectomies.

## 1. Introduction

Primary urothelial cancer of the bladder is a serious worldwide health risk, commonly affecting the elderly and smokers. Radical cystectomy is the standard treatment for muscle-invasive tumors and some cases of high-risk, non-muscle-invasive tumors [1]. Until recently, open radical cystectomy (ORC) via laparotomy was the standard approach. Robot-assisted radical cystectomy (RARC) was introduced in 2003 with the aim of reducing the significant perioperative morbidity of this procedure [2].

Initially, robot-assisted cystectomy was performed with an extracorporeal urinary diversion (eRARC). In this technique, only the removal of the bladder and the lymph node dissection is robot assisted. After completing these steps, the robot is undocked, and a 15–20 cm section of the terminal ileum is exposed through a subumbilical incision. This section is prepared to be used as part of the artificial urinary diversion. The ureters are then anastomosed to one end of the prepared section of the ileum, and the other end of the ileum is sutured to the skin in the lower abdominal area [3].

A meta-analysis of five prospective trials comparing the perioperative outcomes of eRARC versus those of ORC showed less blood loss, fewer blood transfusions, and a shorter hospital stay but longer operation times for eRARC compared to ORC [4,5,6,7,8,9]. In addition, a randomized, open-label, phase-3 study by Parekh et al. demonstrated that eRARC was non-inferior to ORC in regard to 2-year progression-free survival [9].

Recently, there has been a trend of performing urinary diversion totally intracorporeally (iRARC) with the aim to further improve perioperative parameters [10]. Using this technique, the entire reconstruction of the urinary diversion is performed intracorporeally with robotic assistance. This includes bowel division, dissection of the ileum to be used as an ileal conduit, establishing bowel recontinuity, and ureteroileal anastomosis [3,11].

A systematic review and meta-analysis by Katayama et al. found a further reduction in blood loss and transfusion rates on comparing intracorporeal to extracorporeal urinary diversion. The oncological outcome in the meta-analysis was found to be comparable between iRARC and eRARC, while a lower risk for major complications for iRARC in the subgroup of high-volume centers was shown [12]. Furthermore, two other analyses have described a lower rate of gastrointestinal complications with iRARC [13,14].

Despite these findings, one potential disadvantage of this technique is the prolonged learning curve compared to eRARC, due to the challenging nature of the reconstructive part of the procedure [10]. The learning curve is the period during which a particular procedure is more difficult, lengthy, or associated with a higher risk of complications due to the inexperience of the surgeon [15]. Therefore, the learning curve of a surgeon can have a relevant impact on perioperative outcome and morbidity after iRARC. Learning curves have been examined in many surgical procedures, but so far, no generally accepted and validated assessment tool has emerged. Additionally, the thresholds to define a learning curve tend to vary from study to study [16,17].

However, there is a lack of studies evaluating the learning curve of iRARC, as most available data evaluate eRARC or do not include uniform cohorts [17,18]. Therefore, we describe the learning curve of one single surgeon uniformly using the iRARC technique with urinary diversion via ileal conduit in a retrospective cohort of patients treated with cystectomy.

## 2. Materials and Methods

### 2.1. Study Population and Elegibility

Patients who were treated with cystectomy at our institution from May 2017 to December 2021 were identified, and a retrospective chart review was conducted. Patients who underwent cystectomy for reasons other than primary malignancy of the urinary bladder, such as bladder-invasive colorectal cancer or neurogenic bladder dysfunction, were excluded from the study. A documented rejection of a patient to contribute personal data for research purposes also led to patient exclusion. Only patients receiving intracorporeal reconstruction of the urinary tract via ileal conduit as urinary diversion were included.

### 2.2. Surgeon and Surgical Technique

The first surgeon (A.S.) to perform iRARC at our institution had extensive prior experience in open surgery, including ORC. He had been performing robot-assisted procedures, such as prostatectomy, for five years before starting to adopt robot-assisted cystectomy. For the first three iRARCs performed at our center, an experienced mentor was present in the operating room to provide teaching and assistance. Cystectomies performed by other surgeons were excluded. All assistant surgeons involved were certified urologists and highly experienced consultants.

The technique used for robotic radical cystectomy at our clinic is similar to the method described by Jonsson et al. at Karolinska Institutet [11]. After docking the robot, the ureters are identified and dissected. The bladder is dissected and removed, and the left ureter is tunneled under the sigmoid mesentery to the right side. Additionally, an extended pelvic lymph node dissection is performed. Following this, a 20 cm section of intestine is isolated from the terminal ileum, and the ureteroenteral anastomosis is constructed using the Wallace technique over two ureteral stents. After 10 days, urinary leakage is excluded via conduitography under antibiotic prophylaxis. The stents on both sides are removed on day 11 and 12, and patients are usually discharged on day 14. During the first 47 operations, the DaVinci Si system was used, while for patients 48–53, the DaVinci Xi system was utilized. However, the surgical technique remained unchanged throughout the entire learning curve.

### 2.3. Study Data

Demographical, clinical, pathological, and perioperative data were collected from the institutional clinical information system. Blood loss was calculated by subtracting the amount of irrigation used from the volume in the suction canister. The data acquisition was carried out by the first author (C.A.).

The staging was based on the TNM classification from the American Joint Committee on Cancer [19]. The American Society of Anesthesiology (ASA) classification system and the Charlson comorbidity index (CCI) were used to report preoperative comorbidities.

Complications that occurred intraoperatively and within 90 days postoperatively were recorded, respectively. The Clavien system was used to measure the severity of postoperative complications [20]. The complication with the highest Clavien–Dindo index category was used for perioperative outcome analysis. Minor complications were defined as Clavien–Dindo index grade I-II, and major complications were defined as Clavien–Dindo grade III-V. The criteria for urogenital infection included treatment with antibiotics, a positive urine culture, and one of the following findings with no other cause: elevated infection parameters, fever, or typical symptoms. Pyelonephritis was defined as a urinary tract infection with flank pain or tenderness in the loin. According to these criteria, these urinary infections were categorized as grade II. Routine antibiotic prophylaxis during conduitography and the removal of the DJ catheters were not considered as complications. Paralytic ileus with gastric tube insertion was defined as CDI grade I, and parenteral nutrition as CDI grade II. Interventions for which local or general anesthesia was required were classified as grade III. Procedure-specific complications, such as uretero-ileal obstruction or urinary leakage, were included. As described by Clavien et al., all postoperative complications were included for analysis, even if not related to surgery with certainty [21].

Follow-up data for a total duration of 90 days were collected from our clinical information system. Documentation from patients who were not followed at our institution but in affiliated practices was obtained and saved in our clinical information system. No patients were lost to follow up during the 90-day period. To the best of our knowledge, the dataset is complete, and no relevant data are missing.

### 2.4. Study Objectives

The aim of the study was to analyze the association between surgical experience and perioperative outcome. Surgical experience was defined as the number of operations performed prior to the current operation. The patient cohort was subdivided into four groups of equal size based on increasing surgical experience. The primary endpoints were operation time, intraoperative blood loss, duration of hospitalization, total lymph node yield, and perioperative complications.

### 2.5. Statistical Analysis

The categorical variables were reported using absolute and relative frequencies. For the comparison of relative frequencies among the four surgical groups, Fisher’s exact test was used. Continuous variables were reported using the median and the interquartile range. For the comparison of baseline variables, the Kruskal–Wallis test was used. To compare the medians of continuous outcome variables, the Jonckheere–Terpstra test was used, which compares medians and tests for trends across the groups. The medians of directly consecutive groups were compared using the Mann–Whitney–Wilcoxon test. Multivariate regression models were used to identify independent predictors for perioperative outcome parameters.

To investigate the association between outcome parameters and surgical experience as a continuous variable, logistic regression and linear regression were conducted for categorical and continuous variables, respectively. This continuous analysis did, therefore, not involve the four surgical groups. For all statistic models, the tests were two-sided and *p* ≤ 0.05 was considered significant, without correction for multiple testing. For graphical representation, conventional tables, bar charts, and boxplots were used. The statistical analyses were performed using R software (version 4.2.2) [22].

## 3. Results

### 3.1. Patient Characteristics

We identified 97 patients who underwent radical cystectomy for bladder cancer at our institution between May 2017 and December 2021. Two patients refused to contribute personal data for research purposes. Moreover, 18 patients receiving ORC, 7 patients receiving other urinary diversions than an ileal conduit such as a neobladder or a ureterocutaneostomy, and patients not operated on by A.S. were excluded (Table A1 in Appendix A).

In all, 53 patients receiving RARC by A.S. with an ileal conduit as the urinary diversion were included for the learning curve analysis. They were divided into four groups of equal size in ascending order of surgical experience. All the 18 excluded open cystectomies were performed during the time period of the first two groups. During the timespan of groups 3 and 4, no open cystectomies were concurrently performed. During the first two groups, all RARCs were performed by A.S., whereas after July 2020, a second surgeon was trained. Those cystectomies were, therefore, excluded from the learning curve analysis. All operations planned as iRARCs with ileal conduit were successfully completed without the need for conversion. The demographical and clinical data are detailed in Table 1. The median age at operation was 72 years. The groups were homogeneous in terms of age (*p* = 0.8, median 72 years), sex distribution (*p* = 0.2, 17% female sex), body mass index (*p* = 0.3, median 25.7 kg/m^2^), ASA class (*p* = 0.8, 51% class III, 0% class IV), and Charlson comorbidity index (*p* = 0.8, 68% CCI = 0). Exposure to previous treatments did not differ significantly, 11% of patients received neoadjuvant chemotherapy. All patients underwent preoperative local staging using CT scans.

### 3.2. Pathological Characteristics

The pathological characteristics are shown in Table 2. Overall, 77% of pathologic specimens showed muscle-invasive disease. The T stage differed significantly among groups (*p* = 0.04). No non-muscle-invasive tumors were found in group 1, while more advanced tumors (>pT3) were operated on in group 3 than in the other groups. The pathological lymph node status was comparable (pN1-2 in 23%), while positive surgical margins were more frequently found in group 3 (31% vs. 8% or lower, *p* = 0.02).

### 3.3. Operation Time

**Figure 1:**
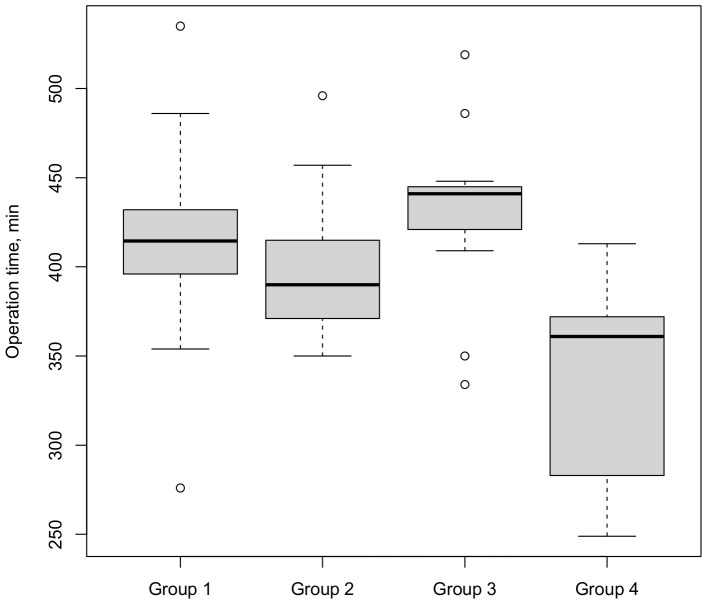
Operation time stratified by four consecutive surgical groups (○ = outlier).

The patients’ perioperative characteristics are presented in Table 3. The median operation time (skin to skin) was 396 min and showed a significant decline over the entire observation period (Figure 1), from 415 to 361 min (*p* = 0.02), with a peak in third group (441 min). After this peak, the operation time declined significantly between group 3 and group 4 (*p* < 0.001, Table A2). Continuous analysis of operation time confirmed a decline over time (*p* = 0.01). On multivariate regression analysis (Table 4), surgical experience was found to be an independent predictor of shorter operation time (*p* = 0.04) and a postoperative T4 staging was associated with a longer operation time (*p* = 0.01).

### 3.4. Blood Loss

**Figure 2:**
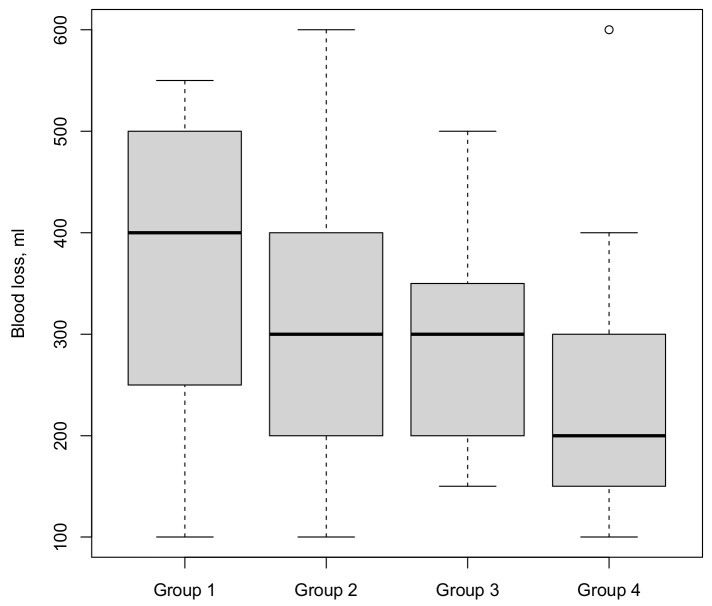
Blood loss stratified by four consecutive surgical groups (○ = outlier).

The median estimated total blood loss (Figure 2) in group 4 (200 mL) was half the amount of group 1 (400 mL; *p* = 0.01). This decline in blood loss was statistically significant also in continuous analysis (*p* = 0.01). On multivariate regression, a higher BMI was a predictor for a higher blood loss (*p* = 0.03).

### 3.5. Lymph Node Yield

**Figure 3:**
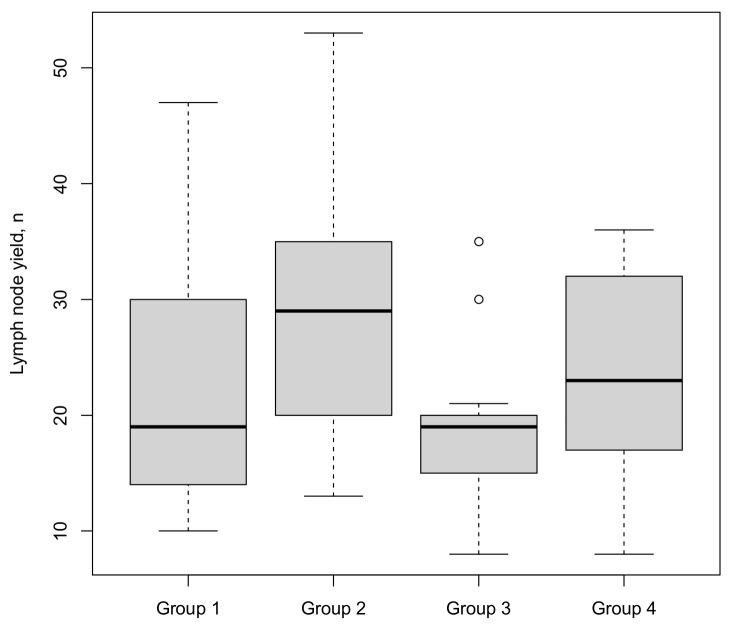
Lymph node yield stratified by four consecutive surgical groups (○ = outlier).

All patients received a standard pelvic lymph node dissection as per the EAU guidelines up to the common iliac bifurcation [1]. The median lymph node yield (Figure 3) was 20. This parameter showed no significant trend in analysis over the entire learning curve. No parameters were associated with lymph node yield in multivariate analysis.

### 3.6. Length of Hospital Stay

**Figure 4:**
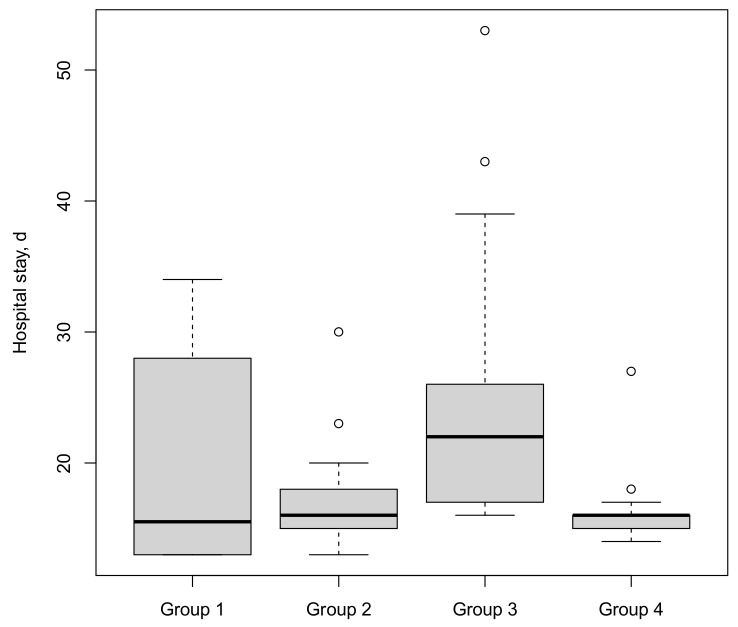
Hospital stay stratified by four consecutive surgical groups (○ = outlier).

The median LOS was 16 days (Figure 4). There was no trend over time in median LOS. The patients in group 3 stayed a median of 6 days longer than the patients in all other groups. Multivariate analysis showed that a predictor of a longer hospital stay was higher age (*p* = 0.03).

### 3.7. Complications

**Figure 5:**
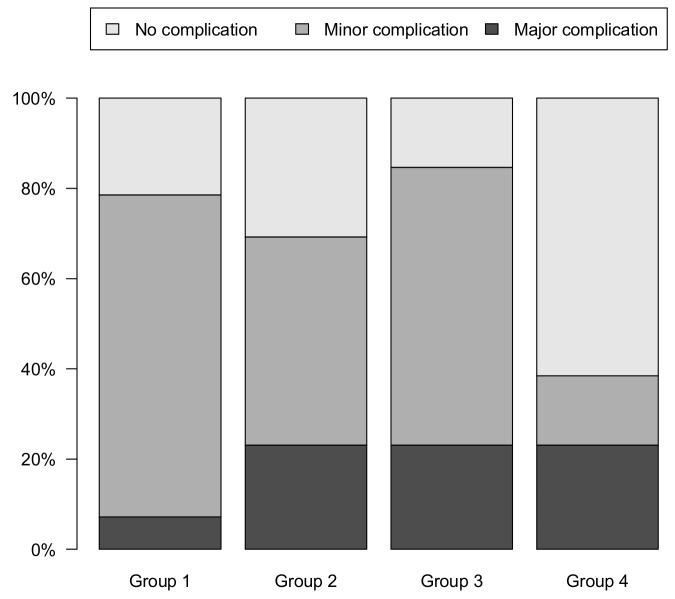
Complication rate stratified by four consecutive surgical groups.

The median overall complication rate was 68%. Overall, 49% of patients had minor complications, while, in 19% of cases, major complications occurred (Figure 5). No patients died within 90 days postoperatively (Clavien–Dindo V).

Although we observed some evidence of a reduction in the overall complication rate from 79% to 38%, the differences between relative frequencies did not reach conventional levels of statistical significance (*p* = 0.07 in group comparison). However, there was a significant drop in overall complications between group 3 and 4, from 85% to 38% (*p* = 0.04).

Minor complications showed a clear decline over the course of the study from 71% to 15% (*p* = 0.02 in group comparison, *p* = 0.03 in continuous analysis). A significant drop from 54% to 15% (*p* = 0.03) was seen between group 3 and 4. No significant trend or change in major complication rate was found.

One rectal lesion in group 3 was the only documented intraoperative complication. In this patient, a lesion of the rectum was repaired intraoperatively. A few days later, an anastomotic insufficiency required a laparotomy, rectum resection, and the installation of a protective ileostoma (Clavien–Dindo IV). The stoma was reversed 4 months later.

## 4. Discussion

A radical cystectomy is a complex and challenging surgical procedure associated with significant morbidity. In recent years, many institutions have transitioned from ORC to eRARC and, thereafter, to iRARC. According to data from the International Robotic Cystectomy Consortium (IRCC), the use of iRARC among member institutions increased from 9% in 2005 to 97% in 2015 among 2125 included cases. However, the demanding technique of iRARC and the steep learning curve is still a major challenge and subject of ongoing debate among robotic surgeons [10].

In 2015, an expert panel of urologic surgeons published the Pasadena Consensus, a set of best-practice recommendations for robot-assisted cystectomies. The panel concluded that, on average, 30 cases were required to achieve proficiency in RARC, regardless of the technique used. However, most of the included patients were operated on using eRARC [18].

Morozov et al. recently published a systematic review of 17 reports on the learning curve analysis of robot-assisted cystectomies, comparing a wide range of perioperative parameters [17]. However, the analysis had several limitations, such as the high degree of heterogeneity among the included studies, the lack of standardized assessment tools to evaluate a surgeon’s learning curve, the limited sample size in certain studies, and the inclusion of multiple surgeons in some case series. In other reports, different forms of urinary diversion such as neobladder and ileal conduit were combined in the same analysis. A majority of the included studies used extracorporeal urinary diversion (eRARC).

Wijburg et al. published a retrospective multicenter analysis of the learning curve in iRARC among nine high-volume hospitals with ≥100 cases. The participating centers in this study used varying proportions of urinary diversion via ileal conduit and neobladder, and in 77% of the centers, more than one surgeon was included in the analysis of a single learning curve [23].

At our institution, the standard operation technique for radical cystectomy was changed from ORC to iRARC starting in May 2017. In the following years, robot-assisted cystectomies were performed by one single surgeon. In all cases, the reconstruction was performed intracorporeally (iRARC). The vast majority of urinary diversions were ileal conduits. This offered the opportunity to analyze a case series of the first 53 patients operated on by one single surgeon, uniformly receiving RARCs with intracorporeal urinary diversion via ileal conduit.

Different perioperative parameters are commonly used to assess the learning curve of surgical procedures. Morozov et el. found that the operation time was the most frequently used parameter in their systematic review. It decreased in almost every study in which it was compared. Between 9 and 50 procedures were required to reduce the operation time significantly [17].

A multivariate regression analysis of our data demonstrated that increasing surgical experience correlated with a shorter operation time. After 40 patients, the median operation time dropped to 361 min, which is comparable to the median operation time of the IRCC cohort (357 min, *n* = 1094) [10].

Even though the operation time showed a significant decline over the entire cohort, an outlier peak of the median 441 min was seen in group 3. In-depth analysis of the patient data revealed a possible selection bias for the time period of the first two groups. The Pasadena consensus suggested that more difficult cases, such as those with bulky tumors or obese patients, should not be operated on during the beginning of the learning curve [18]. Accordingly, during the time in which the first 27 patients (group 1 and 2) received iRARC, 18 patients with either extensive local disease, considerable comorbidities, or other challenging factors were operated on via ORC (excluded in our analysis, as shown in Table A1). In contrast, from group 3 onward, even the more challenging cases were all operated on with robotic assistance. As a consequence, more T4 tumors were operated on in group 3 than in any other group. Multivariate regression analysis identified the T4 stage as an independent predictor of longer operation time.

The length of hospital stay and the overall and minor complications showed an equivalent peak in group 3. After patient 40, these respective parameters decreased significantly, reflecting the surgeon’s increased familiarity with iRARC, even in challenging cases.

The association between surgical experience and blood loss during RARC is controversial. A few studies have shown a decrease in blood loss during training, while others have found no change over time [17]. The reported amount of median blood loss ranges between 200 and 500 mL [10,12,17]. The documented amount is often an estimation and is not measured precisely. At our center, the median blood loss decreased from 400 mL to 200 mL, indicating an association with surgical experience. Multivariate regression analysis also indicated an increased risk for blood loss in obese patients. A higher risk of perioperative bleeding for patients with BMI < 18.5 or >30 kg/m^2^ was previously demonstrated by Lenardis et al. [24].

The average lymph node yield in most publications is between 15 and 30, and optimization is reached after 20–50 cases, although a clear association with the learning curve has not been shown in the majority of recent analyses [17]. At our center, no correlation between surgical experience and lymph node yield could be shown. This is likely due to the fact that the surgeon had already gained ample experience in lymph node dissection during preceding robot-assisted prostatectomies. Thus, no further improvement could be documented.

The postoperative length of hospital stay slightly decreased in some previous studies after 10–15 patients, while, in our cohort, no reduction was shown throughout the learning curve [17]. Postoperative complications can often lead to a longer hospitalization time, while, on the other hand, a shorter LOS might be an indicator of surgical proficiency. However, there are a number of other variables that can influence LOS. In 2019, a large population-based study (*n* = 2448) showed that LOS after cystectomy is more dependent on patient-related factors like age, previous therapies, and comorbidity than on surgical experience [25]. Thus, multivariate regression analysis demonstrated an association of higher patient age with LOS in our data.

Postoperative complications are one of the most heterogeneous measures of surgical experience [17]. Various classification systems have been proposed, with the Clavien–Dindo system being one of the most commonly used [26]. However, even when using the same classification system, interobserver variability introduces potential bias in the classification of complications, making the comparison of data across studies more difficult [21,27].

Most studies analyzed by Morozov et al. only assessed the overall complications. A decline after a certain number of cases was shown in some cohorts, although the exact number varies widely between 10 and 75 cases [17]. Two studies conducted a more detailed analysis of complications, categorizing them by severity level: Dell’Oglio et al. performed an analysis of Clavien–Dindo ≥2 complications and described a protective effect of surgical experience [28]. Porreca et al. analyzed high-grade complications (CDI ≥ 3) and also found a reduction in the incidence during the learning period [29].

Our data showed a linear decline in minor complications over the entire learning curve as seen in the logistical regression analysis, and a significant drop in overall and minor complications after patient 40. In addition to the surgeon’s skill in performing the procedure, the rising experience of other members of the perioperative team, such as nurses, anesthesiologists, and physicians responsible for postoperative care, could also play a role in reducing complications.

Positive surgical margins are a strong predictor of recurrence and shorter cancer-specific survival. It is, therefore, of great importance to achieve a complete resection. As reported in a study by Novara et al., the proportion of PSM among patients increases as the pathological T stage advances, with 1.8% PSM among patients with postoperative pT1 staging, 2.3% for pT2, 7.6% for pT3, and 24% for pT4 tumors [30]. The association of T4 stage with positive surgical margins was confirmed in the multivariate regression analysis in our cohort.

This study is not without limitations. This analysis only registered short-term perioperative outcomes during the first 90 days after surgery and does not provide follow-up data on long-term complications or oncological outcome. It only included patients operated on by one single surgeon with ileal conduit as the urinary diversion, which leads to a limited generalizability. The size of the groups being compared in the study was arbitrary, which may limit the validity of using a threshold of 40 completed cystectomies to indicate proficiency in performing the procedure safely in more complex cases. However, an effect of increasing experience on operation time, blood loss, and minor complications could still be demonstrated when using surgical experience as a continuous variable, independent of the four consecutive surgical groups.

The sample size of 53 cases is relatively small when compared to other studies, such as the work by Wijburg et al. [23]. In contrast, in our analysis, the data of only one surgeon and one kind of urinary diversion were included. Based on the available case numbers, it was not possible to clearly identify a plateau for the investigated parameters. It is possible that a plateau could be reached, as suggested by the research by Wijburg et al., between 100 and 200 cases. However, toward the end of our study, another surgeon started performing cystectomies at our center, which ended the period where the learning curve of one single surgeon could be observed.

Another limitation is the switch from the DaVinci Si system to the Xi system after the 47th patient. Although the surgeon subjectively reported no difference in the ease or speed of the operation with the new system, it is still possible that this change may have had an impact on perioperative parameters.

Overall, the learning curve in iRARC remains a challenging area to study and quantify. While there is a lack of studies comparing uniform cohorts, this study demonstrated the learning curve of one single surgeon using the same operation technique and urinary diversion for their first 53 cases.

## 5. Conclusions

In conclusion, our analysis demonstrates that operation time, blood loss, and minor complications decrease with increasing surgical experience in iRARC in a cohort of patients with urothelial cancer. The findings support the introduction of iRARC in clinics with preexisting robotic experience while suggesting that technically challenging patients should be operated on by surgeons with a surgical experience of more than 40 iRARC procedures.

## Figures and Tables

**Table 1:** Patients’ demographic and preoperative data stratified by surgical experience.

Variable	Overall *n* = 53	First Group *n* = 14	Second Group *n* = 13	Third Group *n* = 13	Fourth Group *n* = 13	*p* Value
Sex female (%)	9 (17)	3 (21)	2 (15)	4 (31)	0 (0)	0.2
Median age, years (IQR)	72 (66–78)	69 (63–80)	71 (66–72)	73 (66–76)	74 (69–80)	0.8
Median BMI, kg/m^2^ (interquartile range)	25.7 (23.7–29.1)	27.8 (25.2–30.3)	25.4 (23.4–27.5)	25.0 (24.1–28.3)	25.2 (22.9–29.0)	0.3
American Society of Anesthesiology Class (%)						
II	26 (49)	8 (57)	5 (38)	6 (46)	7 (54)	0.8
III	27 (51)	6 (43)	8 (62)	7 (54)	6 (46)	
Charlson comorbidity index (%)						
0	36 (68)	8 (57)	10 (77)	9 (69)	9 (69)	0.8
1	8 (15)	1 (7)	2 (15)	2 (15)	3 (23)	
2	7 (13)	4 (29)	1 (8)	1 (8)	1 (8)	
3	2 (4)	1 (7)	0 (0)	1 (8)	0 (0)	
Previous treatment (%)						
BCG instillations	6 (11)	1 (7)	1 (8)	1 (8)	3 (23)	0.7
Chemotherapy instillation	2 (4)	0 (0)	0 (0)	0 (0)	2 (15)	0.2
Neoadjuvant chemotherapy	6 (11)	3 (21)	3 (23)	0 (0)	0 (0)	0.07
Thereof, CP-based	3 (6)	1 (7)	2 (15)	0 (0)	0 (0)	0.5

BCG = Bacillus Calmette–Guerin, CP = cisplatin.

**Table 2:** Patients’ pathologic data stratified by surgical experience.

Variable	Overall	First Group	Second Group	Third Group	Fourth Group	*p* Value
Pathological tumor stage (%)						
<T2	12 (23)	0 (0)	3 (23)	4 (31)	5 (39)	**0.04**
T2	18 (34)	9 (64)	2 (15)	3 (23)	4 (31)	
T3	17 (32)	3 (21)	7 (54)	3 (23)	4 (31)	
T4	6 (11)	2 (14)	1 (8)	3 (23)	0 (0)	
Pathological lymph node status (%)						
pN0, Nx	41 (77)	10 (71)	12 (92)	10 (77)	9 (69)	0.6
pN1, N2	12 (23)	4 (29)	1 (8)	3 (23)	4 (31)	
Positive surgical margins (%)	5 (9)	0 (0)	1 (8)	4 (31)	0 (0)	**0.02**

Bold numbers reflect statistical significance (*p* < 0.05).

**Table 3:** Patients’ perioperative characteristics stratified by surgical experience. Comparison of relative frequencies and medians among the four groups was performed using Fisher’s exact test ^1^ and Jonckheere–Terpstra test ^2^, respectively. Linear ^3^ or logistic ^4^ regression was used to test for a trend with surgical experience as a continuous variable. ^a^ Statistically significant difference compared to the previous group (*p* < 0.05) as determined by Mann–Whitney U Test (exact *p* values are detailed in Table A2).

	Overall	First Group	Second Group	Third Group	Fourth Group	*p* Value Groups	*p* Value Continuous
Median operation time, min (IQR)	396 (365–432)	415 (397–432)	390 (371–415)	441 (421–445)	361 ^a^ (283–372)	**0.02 ^2^**	**0.01 ^3^**
Median blood loss, mL(IQR)	300 (200–400)	400 (263–500)	300 (200–400)	300 (200–350)	200 (150–300)	**0.01 ^2^**	**0.01 ^3^**
Median lymph node yield, *n* (IQR)	20 (16–31)	19 (14–28)	29 ^a^ (20–35)	19 ^a^ (15–20)	23 (17–32)	0.8 ^2^	0.8 ^3^
Median hospital stay, days (IQR)	16 (15–22)	16 (13–26)	16 (15–18)	22 (17–26)	16 ^a^ (15–16)	0.8 ^2^	0.8 ^3^
90-day complication rate, *n* (%)							
Overall	36 (68)	11 (79)	9 (69)	11 (85)	5 ^a^ (38)	0.07 ^1^	0.1 ^4^
Minor	26 (49)	10 (71)	6 (46)	8 (62)	2 ^a^ (15)	**0.02 ^1^**	**0.03 ^4^**
Major	10 (19)	1 (7)	3 (23)	3 (23)	3 (23)	0.7 ^1^	0.3 ^4^
Intraoperative complication rate, n (%)	1 (2)	0 (0)	0 (0)	1	1 (8)	0.17 ^1^	0.3 ^4^

Bold numbers reflect statistical significance (*p* < 0.05), IQR = interquartile range. 1 = Fisher’s exact test, 2 = Jonckheere-Terpstra test, 3 = linear regression, 4 = logistic regression, a = statistically significant compared to the previous group.

**Table 4:** Multivariable logistic and linear regression analysis predicting outcome parameters operation time, blood loss, lymph node yield, hospital stay, complications, and positive surgical margins.

	Surgical Experience		Age		Body Mass Index		Tumor Stage (T4)	
	OR (95% CI)	*p*	OR (95% CI)	*p*	OR (95% CI)	*p*	OR (95% CI)	*p*
Operation parameters								
Operation time	−1.1 (−2.1 to −0.1)	**0.04**	−1.2 (−2.8 to 0.5)	0.2	2.9 (−1.0 to 6.9)	0.14	62 (14 to 110)	**0.01**
Blood loss	−2.2 (−4.7 to 0.2)	0.08	−1.3 (−5.3 to 2.7)	0.5	10.9 (1.4 to 20.3)	**0.03**	45 (−70 to 159)	0.4
Hospital stay	−0.06 (−0.2 to 0.1)	0.4	0.3 (0.02 to 0.5)	**0.04**	−0.5 (−1.1 to 0.08)	0.09	6.0 (−0.9 to 13)	0.09
Lymph node yield	−0.04 (−0.3 to 0.2)	0.7	0.1 (−0.2 to 0.5)	0.4	−0.2 (−1.0 to 0.6)	0.6	2.3 (−7.2 to 12)	0.6
Positive surgical margins	1.1 (1.0 to 1.4)	0.2	1.0 (0.8 to 1.4)	0.8	1.1 (0.7 to 1.6)	0.63	754 (18 to 2′227′969)	**0.01**
Complication rate								
Overall	1.0 (1.0 to 1.1)	0.2	1.1 (1.0 to 1.2)	0.1	1.1 (0.9 to 1.3)	0.5	0.6 (0.03 to 4.7)	0.7
Minor	1.0 (0.9 to 1.0)	0.08	0.9 (0.8 to 0.98)	**0.02**	1.1 (0.9 to 1.3)	0.4	0.8 (0.1 to 5.1)	0.8
Major	1.0 (1.0 to 1.1)	0.7	1.1 (1.0 to 1.2)	0.2	0.8 (0.6 to 1.0)	0.1	2.6 (0.3 to 21)	0.4

OR = odds ratio. CI = confidence intervals. Bold numbers reflect statistical significance (*p* < 0.05).

## Data Availability

The data presented in this study are available upon request from the corresponding author. The data are not publicly available due to privacy restrictions.

## Appendix A

**Table A1:** Included patients stratified according to four consecutive groups of patients.

	Total	Group 1	Group 2	Group 3	Group 4
Operation date		01.05.2017–12.03.2019	02.04.2019–11.11.2019	12.11.2019–02.07.2020	30.07.2020–31.12.2021
Total Cystectomies	95	31	17	19	28
Excluded ORC	−18	−14	−4	0	0
RARC	77	17	13	19	28
Excluded neobladder	−4	−3	0	0	−1
Excluded ureterocutaneostomy	−3	0	0	−3	0
RARC, Ileum conduit	70	14	13	16	27
Excluded other surgeon	−17	0	0	−3	−14
RARC, Ileum conduit, ASA	53	14	13	13	13

**Table A2:** Comparison of consecutive groups using Mann–Whitney U test and Fisher test.

Variable	1 vs. 2	2 vs. 3	3 vs. 4
Median operation time (min)	0.1	0.06	**<0.001**
Median blood loss (mL)	0.3	0.8	0.1
Median lymph node yield, *n* (range)	**0.049**	**0.01**	0.1
Median hospital stay, days	0.68	**0.02**	**0.002**
90-day complication rate, *n* (%)			
Overall	0.7	0.7	**0.04**
Minor	0.3	1	**0.04**
Major	0.3	1	1

Bold numbers reflect statistical significance (*p* < 0.05).
